# Three port logic gate using forward volume spin wave interference in a thin yttrium iron garnet film

**DOI:** 10.1038/s41598-019-52889-w

**Published:** 2019-11-11

**Authors:** Taichi Goto, Takuya Yoshimoto, Bungo Iwamoto, Kei Shimada, Caroline A. Ross, Koji Sekiguchi, Alexander B. Granovsky, Yuichi Nakamura, Hironaga Uchida, Mitsuteru Inoue

**Affiliations:** 1 Department of Electrical and Electronic Information Engineering, Toyohashi University of Technology, 1-1 Hibarigaoka, Tempaku, Toyohashi, Aichi 441-8580 Japan; 2 JST, PRESTO, 4-1-8 Honcho, Kawaguchi, Saitama, 332-0012 Japan; 3 Department of Materials Science and Engineering, Massachusetts Institute of Technology, 77 Massachusetts Avenue, Cambridge, Massachusetts, 02139 USA; 4 Faculty of Engineering, Yokohama National University, 79-5 Tokiwadai, Hodogaya, Yokohama, Kanagawa 240-8501 Japan; 5 Faculty of Physics, Moscow State University, Leninskie Gory, Moscow, 119992 Russia; 6 Department of Physical Electronics and Technology, St.Petersburg Electrotechnical University, St. Petersburg, 197376 Russia

**Keywords:** Electronic devices, Electrical and electronic engineering

## Abstract

We demonstrate a logic gate based on interference of forward volume spin waves (FVSWs) propagating in a 54 nm thick, 100 μm wide yttrium iron garnet waveguide grown epitaxially on a garnet substrate. Two FVSWs injected by coplanar waveguides were made to interfere constructively and destructively by varying their phase difference, showing an XNOR logic function. The reflected and resonant waves generated at the edges of the waveguide were suppressed using spin wave absorbers. The observed isolation ratio was 19 dB for a magnetic field of ~2.80 kOe ( = 223 kA m^−1^) applied perpendicular to the film. The wavelength and device length were ~8.9 μm and ~53 μm, respectively. Further, the interference state of the SWs was analyzed using three-dimensional radio frequency simulations.

## Introduction

Wave-based devices enable non-Boolean operations and are attractive for next-generation information processing. Magnonic circuits based on spin waves (SWs)^1–8^ show great potential in this regard because of their low Joule heating as well as the wide range of tunability (>1 mm) of the wavelength of SWs, which can be varied by changing the waveguide and/or the antenna design. The footprint of devices based on SW interference can be much smaller than that of existing the radio frequency (RF) devices because of the sub-millimeter wavelength of SWs.

SWs have already been used successfully for forming logic gates to produce two-^9,10^, three-^11–13^, and four-port^14,15^ devices. These devices showed NOT, XOR, XNOR, NAND and NOR logic gate functionalities. The functions of the four-port NAND and NOR gates were switchable by adjusting the phase of the additional SW input^14^, and additionally, these devices could be used as a three-input majority gate^15^. These devices are based on the interference of electromagnetic waves (EMW)^9,12^ or that of SWs^10,11,13–15^. Furthermore, magnonic crystals^12,16–39^ can be incorporated into SW devices as filters^29,32^ and modulators^26,30,40^. Therefore, all logic gate functions for realizing an arithmetic logic unit have been demonstrated individually so far. The next step in the development of SW integrated circuits (ICs) is combining individual devices to demonstrate concatenation and more complex functionality.

There are three key requirements for the development of SW ICs. First, the SWs should have a large propagation length (at least an order of magnitude longer than the SW wavelength), i.e., the Gilbert damping factor should be low. This propagation length limits the number of logic gates that can be concatenated without amplification. The material with the lowest damping is yttrium iron garnet (YIG, Y_3_Fe_5_O_12_). In comparison, other materials e.g., Heusler alloys^41,42^, permalloy^10,13,43–45^, and CoFeB^46^ have damping at least an order of magnitude higher. However, the preparation of YIG^47–67^ and etching or liftoff processes that preserve the properties of the YIG^60,61,66,68–70^ present challenges, particularly for integration on a semiconductor platform. Second, to efficiently interconnect SW devices, the waveguides need to be curved or bent. This necessitates the use of forward volume (FV) SWs because this is the only SW showing in-plane uniformity^14,71^. The other two SW modes, i.e., backward volume SWs and surface SWs, change their wavelengths when the propagation direction is changed because the angle between the wavevector and magnetic field varies. However, FV SWs suffer from noise generated at the waveguide edge or boundary reflections and strong standing modes^11,71–73^. Finally, when the thickness of the YIG film and the sizes of the antennas are on the order of a millimeter or sub-millimeter, as used in previous logic gates^7,9,11,14,15,74^, the wavelengths of SWs are on a millimeter or sub-millimeter scale resulting in device footprints on the order of mm^2^. Antennas with micron to sub-micron lengthscales can excite short SWs with wavelengths on the similar lengthscales^13,45,59,61,65^. This motivates the development and integration of devices based on thinner YIG films and an antenna with smaller dimensions.

In this study, we have addressed these challenges to successfully demonstrate the smallest three-port XNOR gate to date. The device footprint area was four orders of magnitude smaller than previous work^11^ and the performance was preserved or improved. This is the first integration of a FV SW logic gate using a thin YIG film with device dimensions on the tens of micron scale, and it can be incorporated into further complex circuits because of the freedom in selecting the propagation direction of the FV SW. The improved footprint, wavelength, and isolation ratio were quantitatively compared to previous devices. Further development of device functionalities was analyzed using three dimensional calculations of SW propagation in the fabricated devices modeled on the experimental results.

## Results

### YIG preparation and characterization

All the YIG films used in this study were prepared on 10 mm × 10 mm × 0.5 mm substituted gadolinium gallium garnet (SGGG; [GdCa]_3_[GaMgZr]_5_O_12_) substrates with a (111) orientation using pulsed laser deposition (AOV PMAD-256). The temperature of the substrate during growth was approximately 850 °C under an oxygen pressure of 2.6 Pa while the base pressure was 2 × 10^−4^ Pa. The repetition rate of the pulse laser was 15 Hz, and the growth rate was ~24 nm min^−1^. These values were similar to those used in our previous studies^48,75^.

**Figure 1:**
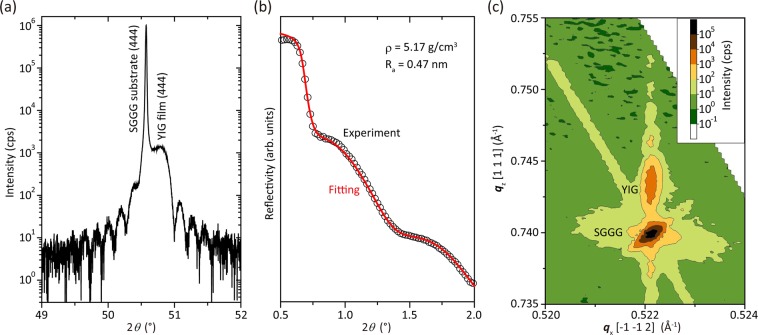
(**a**) 2*θ–ω* XRD of YIG film grown on SGGG substrate. (**b**) XRR of (111)-oriented YIG. (**c**) RSM in the vicinity of the (880) peak.

The 2*θ–ω* X-ray diffraction (XRD) patterns (Fig. 1a) were measured using a Rigaku Smartlab system with a Cu-K(α X-ray radiation source of wavelength 0.15418 nm. The film was fully strained in plane, i.e. the in-plane lattice of the film was matched to that of SGGG substrate, as confirmed by reciprocal space mapping (RSM), Fig. 1c. The YIG unit cell is therefore rhombohedrally strained, and based on the out-of-plane lattice spacing of the (444) reflection and the in-plane lattice match with SGGG, we derived a unit cell side length *A* of 1.2449 nm and corner angle of the unit cell *θ*_CS_ of 90.337°. The unit cell volume of this rhombohedral lattice was 1.9628 nm^3^ ($={A}^{3}\sqrt{1-3{\cos }^{2}{\theta }_{CS}+2\,\cos \,3{\theta }_{CS}}$). These values were similar to those used in our previous studies^76^. X-ray reflectometry (XRR) measurements showed that the films had a smooth surface with an average roughness, *R*_a_, of 0.47 nm and exhibited a density, *ρ*, of 5.17 g cm^−3^ (see Fig. 1b), and a thickness of 54 nm. Composition measurements of a similar film confirmed the Y: Fe ratio was close to 3: 5.

Measurements performed using a vibrating-sample magnetometer (VSM, Tamakawa TM-VSM261483-HGC-SG) indicated that the films had an in-plane coercivity, *H*_c_, of 0.37 Oe, a saturation magnetization, *M*_*s*_ = 143 emu cm^−3^ (4*πM*_s_ = 1800 G), and an out-of-plane saturation magnetic field obtained from a measurement of Faraday rotation angle loop, *H*_*s*_, of 1.73 kOe.

The propagation of SWs in the film was characterized as described in a previous article^76^. Six pairs of antennas with various spacings were fabricated on the YIG film, and transmittance of signals between each pair of antennas was measured using a vector network analyzer (VNA). The measured intensity was plotted as a function of propagation length, yielding the attenuation length *L*_att_ at which the SW intensity decayed by a factor 1/e. The value of *L*_att_ was converted to the net damping parameter α_SW_ which includes the extrinsic damping using^1,76^

1 $${\alpha }_{SW}=\frac{2{({f}_{0}^{2}-{f}^{2})}^{2}(1+\chi )d}{{L}_{att}({f}_{0}^{2}+{f}^{2})f\cdot {f}_{M}(\frac{2}{\chi }-k\cdot d)},$$

where $\chi ={f}_{0}{f}_{M}/({f}_{0}^{2}-{f}^{2})$, ${f}_{0}=-\gamma {H}_{eff}$, ${f}_{M}=-\gamma \cdot 4\pi {M}_{s}$, and ${H}_{eff}={H}_{appl}+{H}_{A}^{Dyn}$. Here *f*, *k*, *d*, *γ*, ${H}_{eff}$, ${H}_{appl}$, and ${H}_{A}^{Dyn}$ represent the frequency, the wavenumber, the YIG thickness, the gyromagnetic ratio ( = 2.8 MHz/Oe for YIG), the effective magnetic field, the applied magnetic field, and the net magnetic anisotropy field obtained from spin wave spectroscopy (SWS, described in the following section). The measured damping factor of the YIG/SGGG for FV SWs was (α_SW_ = ~2^.4^ × 10^−4^ at a frequency of 4 GHz with ${H}_{appl}$ = 2840 Oe, comparable to other reported values (3.0 × 10^−3^ ^62^, 8.79 × 10^−466^ ^4^, × 10^−4^ ^60,64^, 2.8 × 10^−4^ ^63^, 2.3 × 10^−4^ ^65^, 8.0 × 10^−5^ ^61^, 3.6 × 10^−5^ ^49^,).

### Spin wave device preparation

The YIG film was etched into a 400 μm × 100 μm mesa. First, the YIG/SGGG sample was cleaned using acetone, isopropyl alcohol (IPA), and deionized (DI) water with sonication, followed by baking at 180 °C for 3 min. Next, a 300 nm thick layer of a photoresist (ZEON, ZEP520A/ZEP-A = 2: 1) was spin-coated at 2000 rpm and baked at 180 °C for 3 min. An antistatic layer consisting of a conductive polymer (Showa Denko, ESPACER 300Z) was spin-coated at 2000 rpm and baked at 60 °C for 10 min. The sample was then exposed using an electron beam system (EB, JEOL, JBX-6300FS) at a dose of 68 μC cm^−2^. Subsequently, the antistatic layer was removed using DI water. Next, the resist was developed (ZEON, ZED-N50) for 80 s, and the sample was subsequently rinsed with IPA and baked at 120 °C for 5 min. A ~50 nm thick SiO_x_ mask was deposited on the sample by a RF ion-beam sputtering (IBS, OSI-WAVE RM17-0010) at a deposition rate of ~2.6 nm min^−1^. The sample was then etched with phosphoric acid at 140 °C, with the etching process being controlled using an oil bath. The etching rate of the YIG film was ~1.7 nm s^−1^. The remaining SiO_x_ was removed by buffered hydrofluoric acid at an etching rate of ~1.8 nm s^−1^.

**Figure 2:**
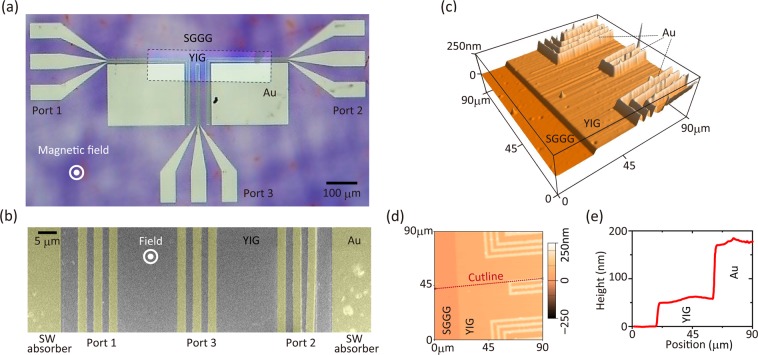
(**a**) Micrograph of FV SW interferometer fabricated using a 54 nm thick YIG waveguide. SW absorbers were fabricated at the edges of the waveguide. The YIG was magnetized out of plane. (**b**) SEM image of the interferometer. (**c**) AFM image of the interferometer. (**d**) Top view of the AFM image showing a cutline as a red dashed line. (**e**) Height profile along the cutline shown in (**d**).

On this YIG waveguide, three antennas (coplanar waveguides) composed of 90 nm thick Au/10-nm thick Ti layers were fabricated using the same resist and a liftoff process. The remaining Au/Ti was removed (ZEON, ZDMAC) at 70 °C for 20 min with 30 s sonication. The Ti layer served as adhesion for the Au layer. These films were deposited by direct current (DC) ion beam sputtering (TDY, 98012-RD). The fabricated 50 μm wide contact pads and antenna patterns are shown in Fig. 2a. The scanning electron microscopy (SEM, JEOL, JEM-6700F) image in Fig. 2b shows the SW injection port. The distance between the two edge signal lines was ~53 μm, while the width and gap of each line in the antennas were 2.23 μm and 1.92 μm, respectively. The antennas had a tilt of 1° with respect to the edge of the YIG waveguide, and were shifted by 90 μm from the edge, due to lithographic alignment errors. In addition, the ground line of the port 2 was slightly misaligned. However, we observed SW propagation and interference processes. The effects of this asymmetric and shifted structure are discussed below. The sample surface was also characterized by atomic force microscopy (AFM, Asylum Research, MFP-3D) as shown in Fig. 2c–e. The taper angle of the etched mesa YIG was 15° with respect to the in-plane direction. The tip used in this observation (Olympus, OMCL-AC200TN-C3) had a radius of curvature of 30 nm (details of AFM conditions are shown in the Methods). Figure 2e showed that the height of the YIG part was between 49 nm and 62 nm. These values show agreement with the result of XRR, ~54 nm. Their difference arises from overetching of the SGGG substrate by the phosphoric acid. Therefore, we assumed a 54 nm YIG thickness in this study.

### Calculation of spin wave wavelength

**Figure 3:**
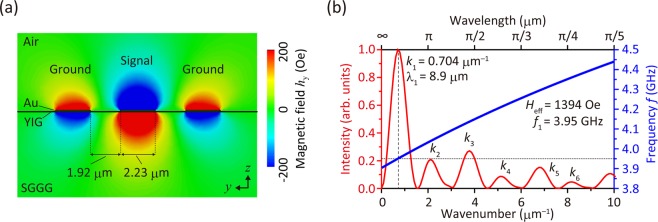
(**a**) Calculated RF magnetic field excited by the antenna used in the experiments. (**b**) Wavenumber spectra generated by the antenna (red line). The strongest peak exhibited wavenumber *k*_1_ of 0.704 μm^−1^, which corresponds to a wavelength *λ*_1_ of 8.9 μm. Dispersion curve of FV SW at *H*_eff_ = 1394 Oe is superposed (blue line), showing an excitation frequency *f*_1_ of 3.95 GHz.

The wavelength of the SWs excited using these antennas was calculated using an RF simulator (CST Microwave Studio 2018) based on the finite integration technique^77,78^. This simulator, which employs Maxwell’s equations, was based on three-dimensional (3D) objects similar to the experimental sample. The calculated RF magnetic field and the propagation of the SWs were in excellent agreement with the experimentally determined values^79^ within the dipolar SW^1^ region. This study treats the SWs as dipolar because *λ*_ex_*k*^2^ «1, where *λ*_ex_ is the exchange constant (3×10^−16^ m^2^ for YIG^1^). Hence, we can ignore the exchange term and use the RF simulator for calculating the SW. Figure 3a shows the calculated EMW excited from the antenna modeled with the same dimensions as the sample used in the experiment. The material parameters used in this simulation are listed in Table 1. The titanium layer was not included in the calculation due to its low thickness.

**Table 1:** Material parameters used in antenna and SW simulations.

Part	Antenna	Film	Substrate
Material	Au	YIG	SGGG
Thickness	100 nm	54 nm	10 μm
Width	2.23 μm	100 μm	300 μm
Gap	1.92 μm	—	—
Conductivity, *σ*	4.561 × 10^7^ S m^−1^	—	—
Permittivity, *ε*_r_	—	15.3	12.1

The distribution of the *y*-component of the RF magnetic field, *h*_y_, obtained at a frequency, *f*, of 3.95 GHz at the bottom of an antenna with a width of 2.23 μm and a gap of 1.92 μm was fast-Fourier-transformed (FFT) into the wavenumber, *k*^65^, as shown in Fig. 3b. The intensity was normalized. The wavelength of the strongest SW (the first mode), *λ*_1_, was 8.9 μm (*k*_1_ = 0.704 μm^−1^). The dispersion curve of the SW was plotted in the same figure using^1,79^

2 $$k=\frac{1}{d\sqrt{\mu }}\,\mathrm{ln}\,\frac{\sqrt{\mu }-1}{\sqrt{\mu }+1},$$

where $\mu =1+{f}_{0}{f}_{M}/({f}_{0}^{2}-{f}^{2})$, ${f}_{0}=-\gamma {H}_{eff}$, and ${f}_{M}=-\gamma \cdot 4\pi {M}_{s}$. Here $\gamma$ = 2.8 MHz/Oe, ${H}_{eff}$ = 1394 Oe, 4π${M}_{s}$ = 1800 G, and *d* = 54 nm. This calculation determined the SW excitation frequency, wavelength, and the required magnetic field. ${H}_{eff}$ was determined as the best fit value that generated the FV SW with *λ*_1_ = 8.9 μm at *f* = 3.95 GHz, in order to match the results of the experiments described below.

### Spin wave spectroscopy

**Figure 4:**
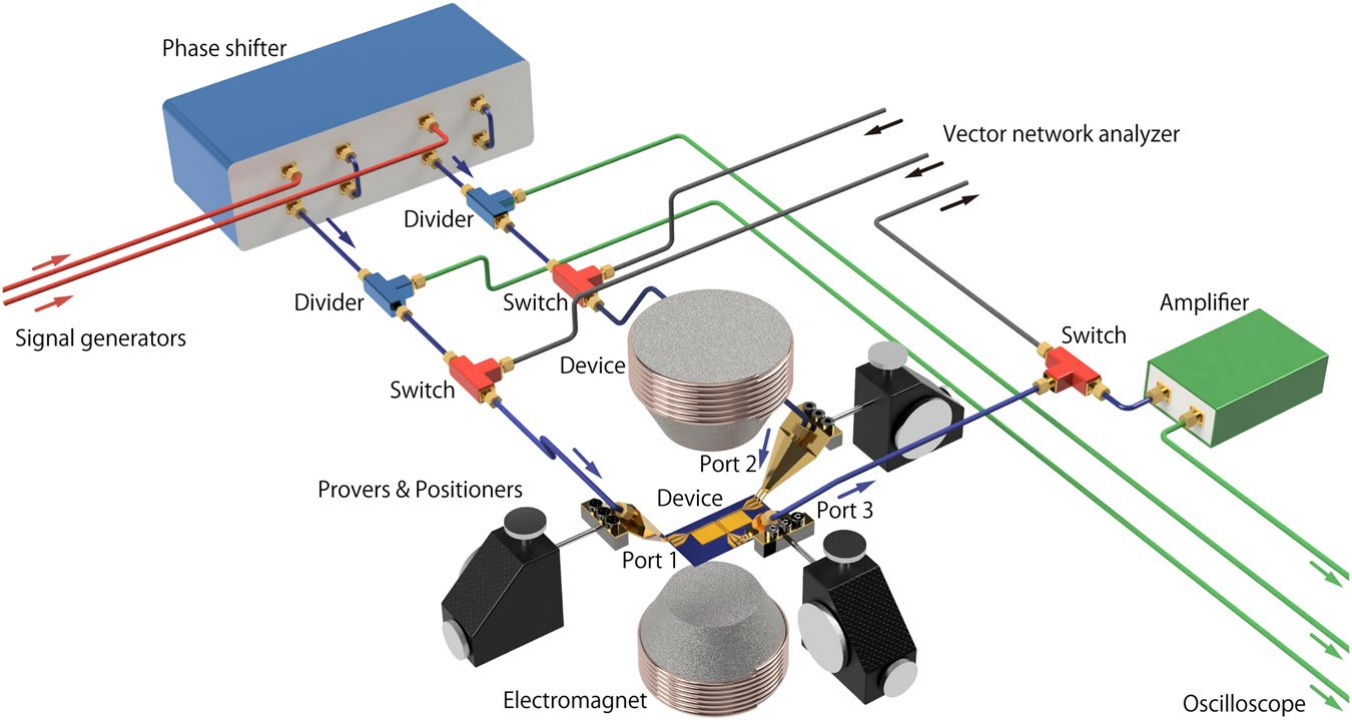
Experimental setup for SWS and SW interference. Three switches change the connection from the probes to the VNA or the oscilloscope.

SW excitation was confirmed and the corresponding wavelengths were determined by SW spectroscopy (SWS) using a vector network analyzer (VNA, Agilent E5071C) and an electromagnet (Toei Scientific Industrial, TKSJ-V500-TYP), shown in Fig. 4. For SWS, three RF switches (Agilent, 3499B) connected the probes to the VNA. The intermediate frequency of the VNA was 100 Hz, and the frequency was varied in steps of 3 MHz. A bias magnetic field was applied perpendicular to the YIG film using an electromagnet under proportional integral differential (PID) control with a Hall probe. The field was varied in steps of 20 Oe. The temperature of the sample was set to 35 °C using a thermostat system (ATTS, A200). The three ports were placed in contact with RF probes (Cascade Microtech, SP-Z40-X-GSG-100) controlled by positioners (Cascade Microtech, RPP210-B-SP-AI).

**Figure 5:**
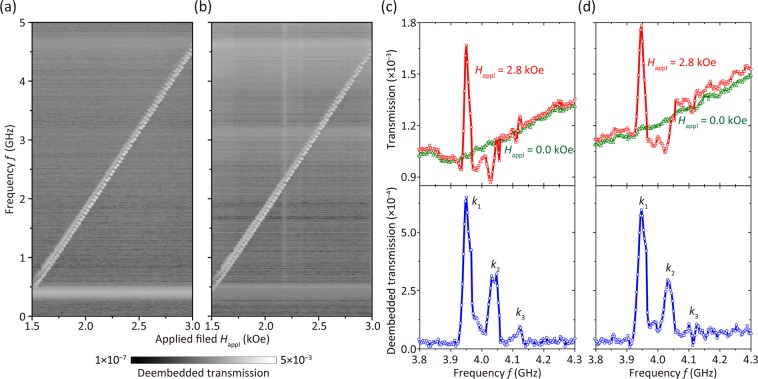
(**a**,**b**) SWS of deembedded transmission from ports 1–3 and ports 2–3, respectively. (**c**,**d**) Transmission from ports 1–3 and ports 2–3 versus frequency, respectively. Red squares and green triangles show data at an applied magnetic field, *H*_appl_, of 2.8 kOe and 0.0 kOe, respectively. Blue circles show deembedded transmission spectra.

Figure 5a,b show the deembedded transmission from port 1 to port 3 and that from port 2 to port 3. SW propagation is displayed as white lines (the broad horizontal line at ~0.5 GHz did not result from SWs). The white lines appear discontinuous because of the 20 Oe step size of the applied field. To extract (de-embed) the transmitted SW, the spectrum at a magnetic field of 0.0 Oe was subtracted from the obtained spectra. This subtraction was conducted for the real part of the transmission from port 1 to port 3 and independently for the imaginary part, and the same was done for the transmission from port 2 to port 3. Examples are shown in Fig. 5c,d. The largest peak corresponds to the first-order SW (*λ*_1_ of 8.9 μm) and was obtained at *f* = 3.95 GHz with *H*_appl_ = 2800 Oe. The positions of the spectral peaks corresponding to *λ*_1_ in Fig. 5c,d were similar, with the difference being 3 MHz. Thus, their coherence is considered high enough for interference. The peak transmission at *H*_appl_ = 2.8 kOe is only ~1.7 times larger than that at *H*_appl_ = 0.0 kOe, which is attributed to the low conversion efficiency of the SW excitation antennas. This could be increased by the use of meander antennas. In Fig. 5a, the intersection of the measured white line and the horizontal axis shows the net magnetic anisotropy field ${H}_{A}^{Dyn}$ = −1346 Oe, the same as in Fig. 5b. Thus, the effective magnetic field ${H}_{eff}={H}_{appl}+{H}_{A}^{Dyn}$ = 2800 − 1346 = 1454 Oe at *f* = 3.95 GHz. This value of *H*_eff_ is 60 Oe larger than that used in the calculation of the dispersion curve shown in Fig. 3b, 1394 Oe. This modest disagreement might be caused by the difference between the ideal models^1^ used in the calculation and the actual sample structure, including thickness variations in YIG observed in the AFM image (Fig. 2e) and/or inhomogeneity of magnetic properties along the thickness direction especially near the boundary between the YIG and the substrate^47,49^.

### Spin wave interference

The two input ports (ports 1 and 2) were connected to the same probe system as that used in the SWS system shown in Fig. 4 and were switched using an RF switch without reconnecting any cables. These input lines were connected to the individual signal generators (Agilent E8257C and HP 83732 A) through power dividers (HP 11636B) and a two-port phase shifter (Colby Instruments PLD-200A-625DS) in order to be able to adjust the difference in the injection phase independently. The divided lines were connected to a digital oscilloscope (Tektronix DSA70804) to monitor the input signals *in situ*. The output port (port 3) was connected to the oscilloscope after amplifying the signal by 30 dB (Amplifier Research, 1S1G4A). All the input voltages were adjusted independently, so that each input voltage was 350 mV. The two signal generators were synchronized using a 10 MHz signal generated by the HP 8647 A generator. The spectral difference between the two inputs was less than 10 Hz.

**Figure 6:**
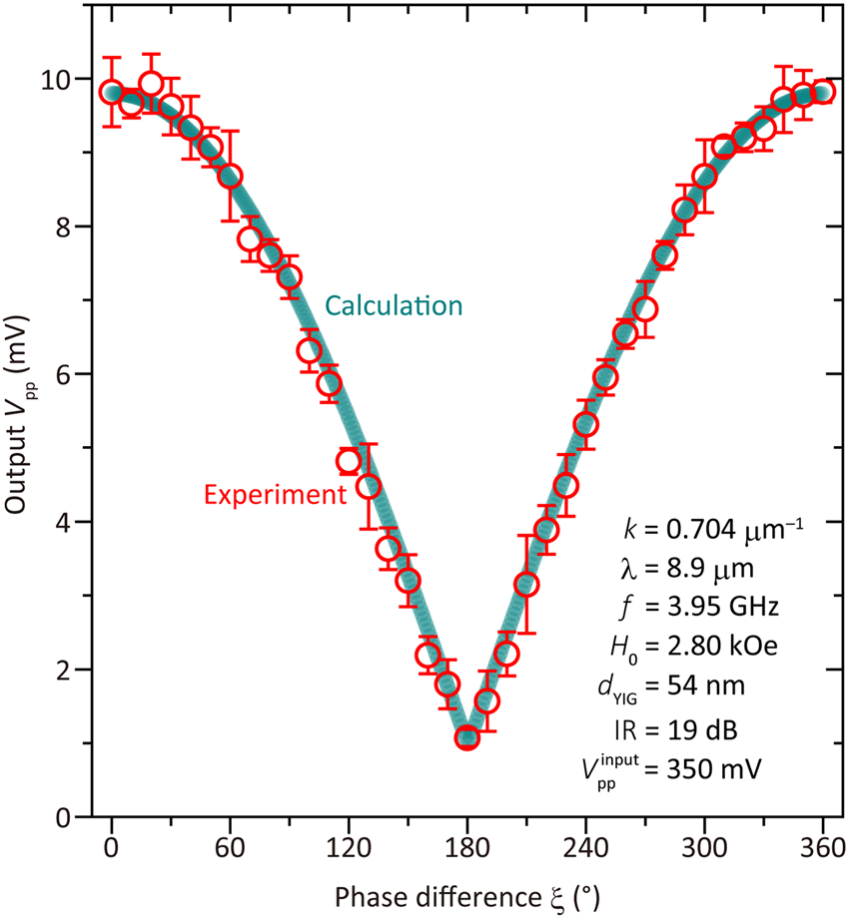
FV SW interference using 54 nm thick YIG waveguide. Red circles show experimental results. Error bars show standard deviation 1σ for five duplicated measurement. Green bold line shows calculated values using Eq. (4).

Figure 6 shows the interference results of the FV SWs at *f* = 3.95 GHz for *H*_appl_ = 2.80 kOe. The interference of the SWs changed as a function of the phase difference, ξ, between ports 1 and 2. This change was analyzed using a fitting technique. The output synthesized wave, *V*, can be expressed as $V=A\,\sin \,\omega t+B\,\sin (\omega t+\xi )$, where *A* and *B* are the amplitudes of the input SWs, *ω* is the angular frequency, and *t* is the time. This was converted to

3 $$V=\sqrt{2}A\sqrt{1+cos\xi }\,\sin (\omega t+\varDelta ),$$

where $\Delta ={\cos }^{-1}\sqrt{(1+cos\xi )/2}$ in the case of *A* = *B*. For the analysis the amplitudes were assumed to be the same for simplicity, even though their amplitudes had ~10% difference as shown in Fig. 5c,d. Therefore, the peak-to-peak voltage was

4 $${V}_{pp}=2\sqrt{2}A\sqrt{(1+cos\xi )}.$$

However, a bias voltage of 1 mV was added as a fitting factor to Eq. (4), and *A* was set to 2.2 mV. Overall, this theoretical line was in good agreement with the experimental results, confirming that constructive and destructive SW interference had indeed occurred.

A phase difference of 0° corresponds to the two input signals (“1”, “1”) or (“0”, “0”). In this case, the output ${V}_{pp}$ is higher than a threshold voltage (e.g. 5 V), and corresponds to an output signal of “1”. In contrast, a phase difference of 180° corresponds to input signals of (“0”, “1”) or (“1”, “0”). In this case, the output ${V}_{pp}$ is lower than a threshold voltage. This corresponds to an output signal of “0”. This behavior represents an exclusive NOR (XNOR) logic gate, expressing the combination of EXOR^80^ and NOT gate.

The maximum and minimum output voltages were 9.80 mV and 1.06 mV, respectively. Thus, the isolation ratio (IR), which measures the on/off performance of the device and is defined as 20 times the log of the ratio of maximum to minimum output voltage, was IR = 19 dB. This was 5 dB larger than that obtained in a previous study of SW interference based on an 18 μm thick YIG waveguide^11^. Moreover, this IR was obtained in a device with a footprint (i.e., the distance between the two edge electrodes) that was 4 × 10^4^ times smaller than that of ref.^11^,at ~4 × 10^−3^ mm^2^. This was possible because of the shortening of the wavelength of the SWs due to the antenna design, and the integration of the antennas on the single-crystalline YIG film. The device performance parameters are listed in Table 2; the results of previous studies are also shown for comparison. The footprint of the XNOR gate, four orders of magnitude smaller than that in ref.^11^, represents an obvious advantage of the integration. Further, the output voltage was an order of magnitude larger than that in the case of permalloy because of the low losses. In addition, IR was no lower than that in the case of bulk YIG, indicating that the fabrication of the SW device did not adversely affect the characteristics of the SW propagation.

**Table 2:** SW device performance observed in this study and those reported in previous works^11,13^.

	54-nm-thick YIG (this study)	18-μm-thick YIG^11^	35-nm-thick permalloy^13^
SW wavelength, *λ*	8.9 μm	780 μm	16 μm
SW wavenumber, *k*	0.704 μm^−1^	8.06 μm^−1^	0.4 μm^−1^
Footprint	4 × 10^−3^ mm^2^	1 × 10^1^ mm^2^	4 × 10^−3^ mm^2^
Distance between input ports	53 μm	10 mm	60 μm
Input voltage	350 mV	200 mV	490 mV
Maximum output voltage	9.80 mV	6.31 mV	0.37 mV
Minimum output voltage	1.06 mV	1.30 mV	0.03 mV
Isolation ratio, IR	19 dB	14 dB	22 dB
Applied field, *H*_0_	2.80 kOe	3.05 kOe	0.44 kOe
Frequency, *f*	3.95 GHz	4.0 GHz	6.5 GHz
SW mode	Forward volume	Forward volume	Surface

### Simulation of spin wave interference

**Figure 7:**
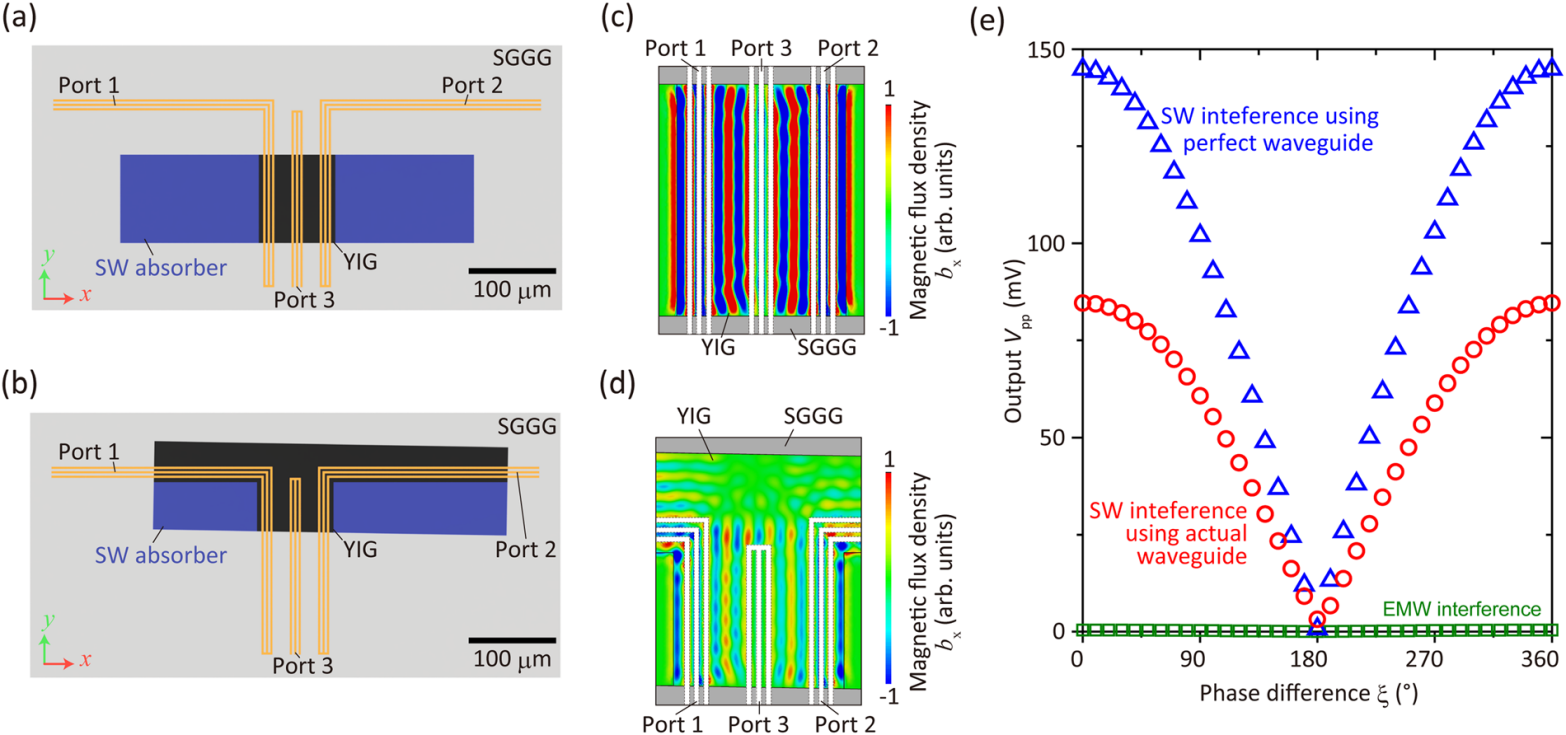
(**a**) 3D model of the interferometer with perfect alignment. (**b**) Model of the actual interferometer with 1° tilt and 90 μm shift between the waveguide and the antenna. (**c**,**d**) The distribution of magnetic flux density *b*_x_ in the vicinity of the output port at the center plane in the thickness direction of YIG in the case of (**c**) perfect waveguide and (**d**) actual waveguide. Phase difference was zero. (**e**) Simulated interference results for perfect waveguide (blue triangles) and actual device (red circles). An asymmetric wave interference state resulted in imperfect destructive interference. These results were all calculated at *f* = 3.95 GHz to see FV SW interference, but the calculation of EMW interference (green squares) was conducted at *f* = 3.80 GHz to eliminate the contribution of SWs.

Although interference was demonstrated successfully in this study, imperfect destructive interference was observed. The remaining voltage in the out-of-phase state was 1 mV. To investigate the cause of this, a finite integration technique simulation was performed on a 3D model of the sample structure, including the tilt angle of 1° and a parallel micron-scale shifting of the antennas on the YIG waveguide, as shown in Fig. 7a,b. The material parameters used were shown in Table 1. SW absorbers were introduced into the model in the areas with high damping (α = 1 × 10^−2^). The damping factor of YIG was taken to be 2.4 × 10^−4^, which is the same as the experimental value. All ports had an impedance of 50 Ω, and were set as the discrete port in the software. A simulation corresponding to the ideal situation, wherein the sample had a completely symmetrical structure, was also performed for comparison, as shown in Fig. 7a.

The results indicated that the asymmetrical interferometer exhibited a larger bias voltage than did the symmetrical one as shown in Fig. 7d. In the case of the symmetric structure, cancellation of the SWs was observed at the center of port 3 (see Fig. 7c). Hence, the output voltage was almost zero, as can be seen from Fig. 7e. In addition, the voltage corresponding to the in-phase state was lower than that for the symmetrical case. This was because of the decrease in the overlapping length of the antenna on the YIG waveguide. In the actual waveguide, only ~62.4% of the antenna covered the YIG waveguide, and the output voltage was approximately 61.9% of that for the ideal case, indicating a quantitative agreement between the two. Obviously, these issues can be prevented by ensuring precise alignment between the antenna layer and the YIG waveguide layer. Therefore, such an improvement should increase both the IR and the robustness of operation of the XNOR logic gate. Supplementally, the interference of EMW was calculated at *f* = 3.80 GHz where SWs cannot be excited. This result shows the output *V*_*pp*_ generated by EMW was three orders of magnitude lower than that of SWs. This is negligible and it can be concluded that the interference of FV SWs was demonstrated successfully in this experiment.

## Conclusion

In this study, a three-port XNOR gate based on FV SW interference at a frequency of ~3.95 GHz was demonstrated, using a 54 nm thick single crystalline YIG waveguide grown on an SGGG substrate and three coplanar waveguides exciting FV SWs with a wavelength of 8.9 μm. The device footprint was 4 × 10^−4^ times that reported previously^11^, with the device showing a 19 dB isolation ratio. The integration of the SW device did not adversely affect the properties of the SWs, which is attributed to the high quality of the fabricated YIG film. The geometry of the SW waveguide was analyzed, and it was concluded that the asymmetric structure arising from fabrication was responsible for the imperfect destructive interference observed. The proposed technique should find use in the development of fundamental logic gates based on thin YIG films. Thus, this study is an important milestone towards the realization of more complex functionality in SW ICs.

## Methods

### Deposition of SiO_x_

The SiO_x_ was coated by RF ion beam sputtering (IBS, OSI-WAVE RM17-0010) at room temperature using a sintered 4 inch SiO_x_ target in 10 mTorr (=1.3 Pa) and 7.5 sccm Ar gas flow and 6.0 sccm O_2_ gas flow with an RF power of 114 W. The base pressure was 6×10^−6^ Torr (=8×10^−4^ Pa).

### Deposition of Au and Ti

A 90 nm thick Au and a 10 nm thick Ti were deposited by DC IBS (TDY, 98012-RD) at room temperature using a 4 inch Au and Ti target. Au was deposited in 3 mTorr ( = 0.4 Pa) pressure and 5.0 sccm Ar gas flow with a DC power of 40 W at a deposition rate of 16 nm min^−1^. Ti was deposited in 20 mTorr ( = 2.7 Pa) pressure and 5.0 sccm Ar gas flow with 50 W power. The deposition rate was 10 nm min^−1^.

### AFM

Specifications and settings of the AFM and its probe used in the experiments were shown in Table 3 and Table 4.

**Table 3:** Specification and setting of the AFM. *AmpinvOLS* and *Defl. InvOLS* show the amplitude inverse optical lever sensitivity, and the deflection inverse optical lever sensitivity, respectively.

	Name/Parameter
Model	Aslyum Research, MFP-3D
Software version	14.30.157 worked with Igor Pro ver. 6.3.7.2.
Scanning area (maximum)	90 μm × 90 μm
Scanning rate	0.2496
Scanning line	256
Imaging mode	Contact
Spring constant	0.03311 N/m
AmpinvOLS	339.86 nm/V
Defl. InvOLS	311.8 nm/V
Integral gain	1.2
Proportional gain	1
Drive Frequency	11.478 kHz
Q value	34.9
Resonance Frequency	11.54 kHz

**Table 4:** Specification of the AFM probe.

	Name/Parameter
Model	Olympus, OMCL-TR400PB-1, series B
Position offset	−4 μm
Shape	Pyramidal
Tip height	2.9 μm
Tip curvature radius	30 nm
Tip material	SiN
Lever material	SiN
Coating material (both side)	Au
